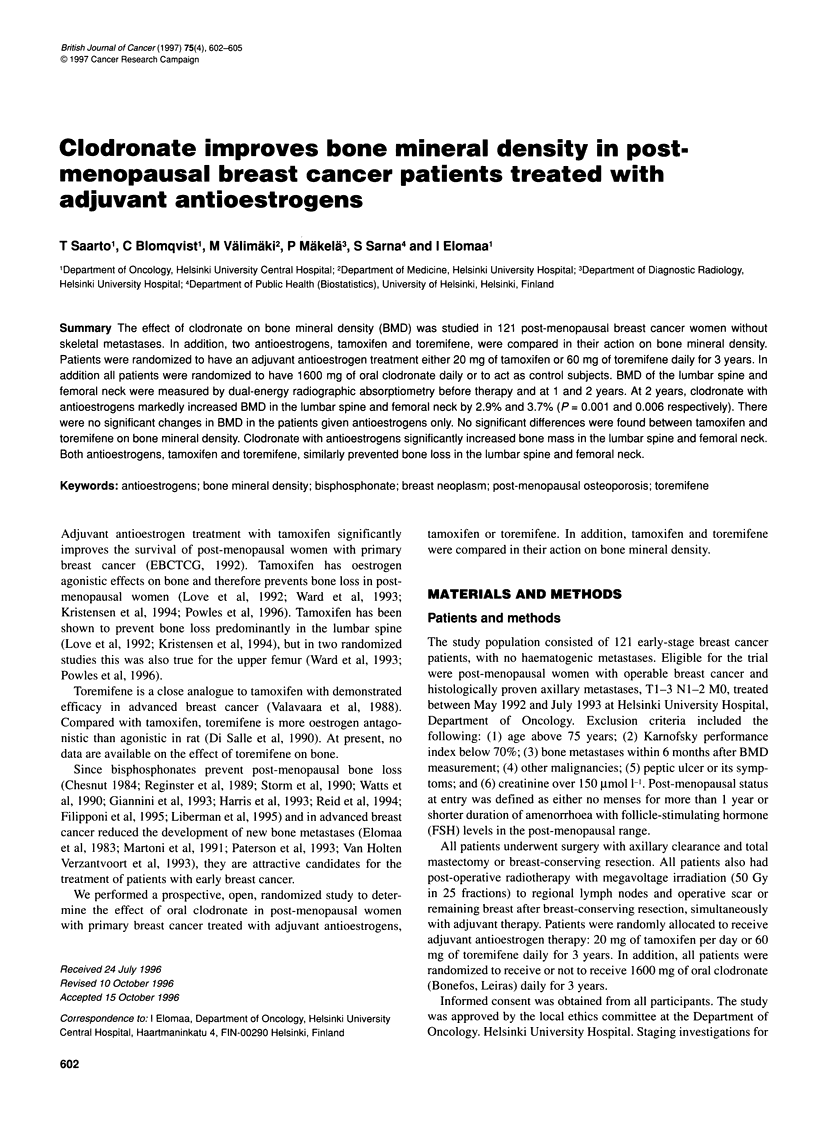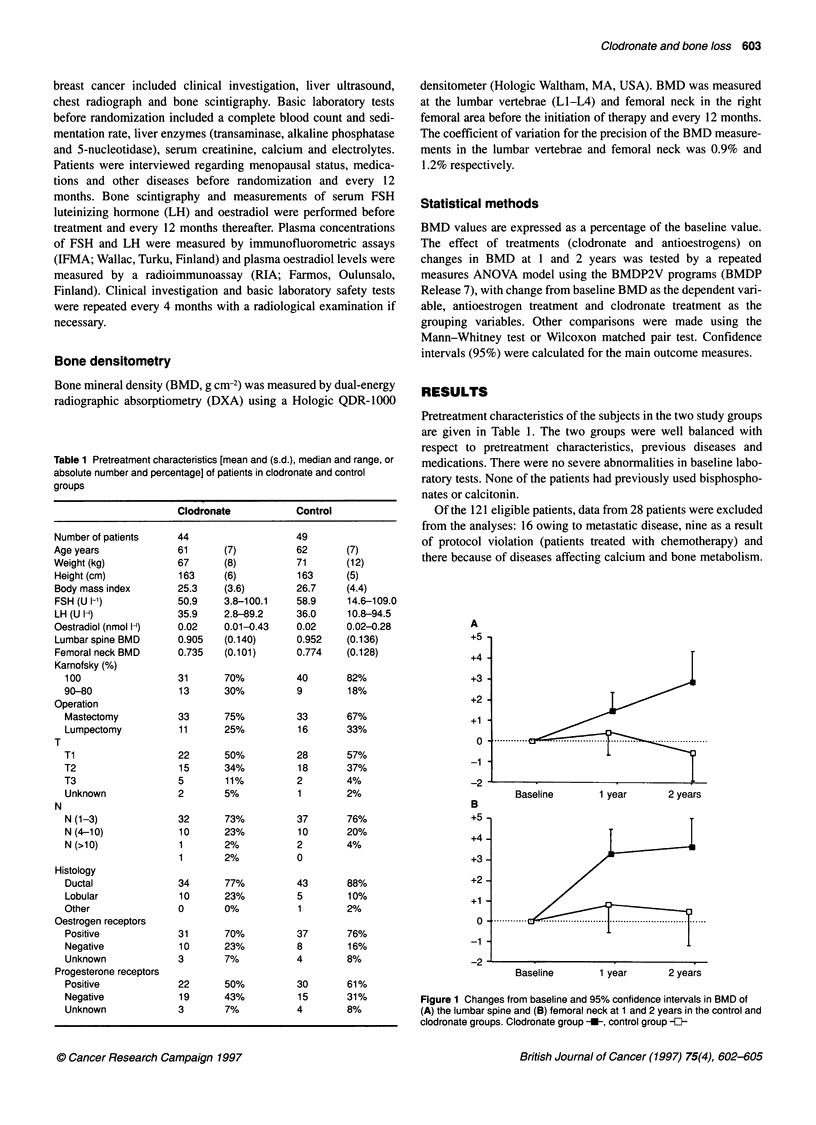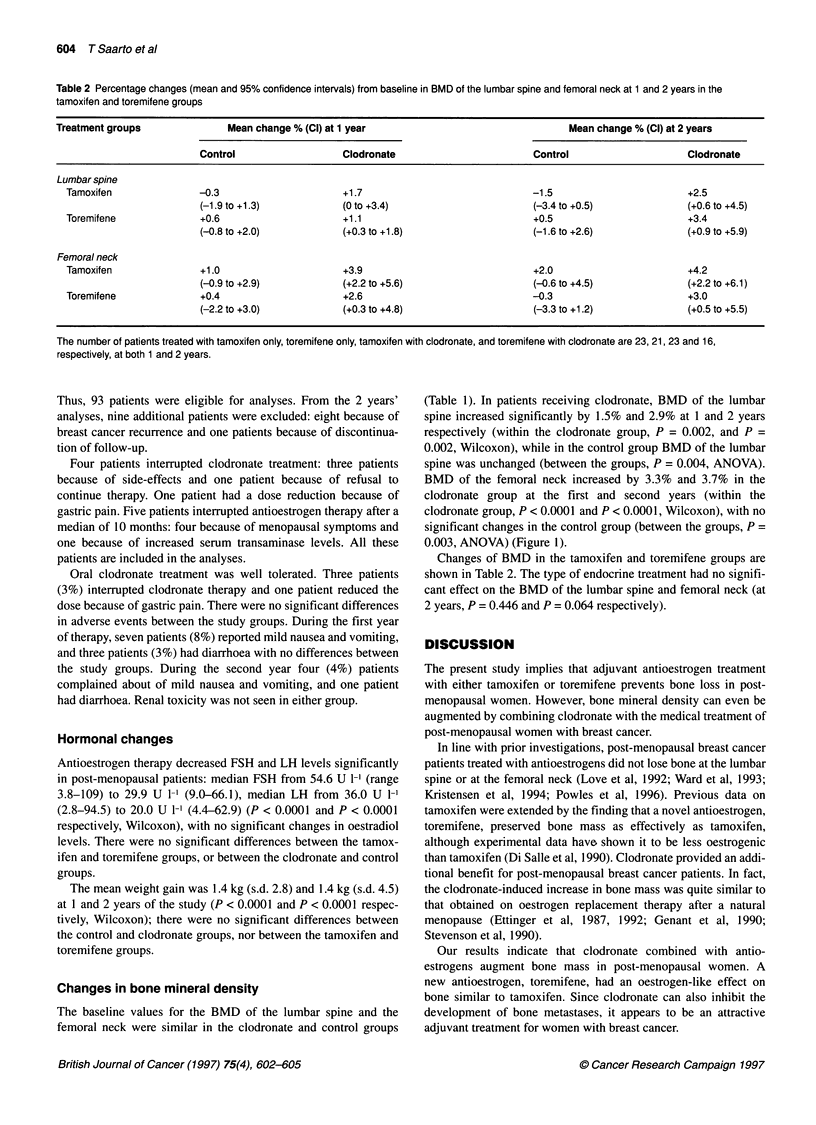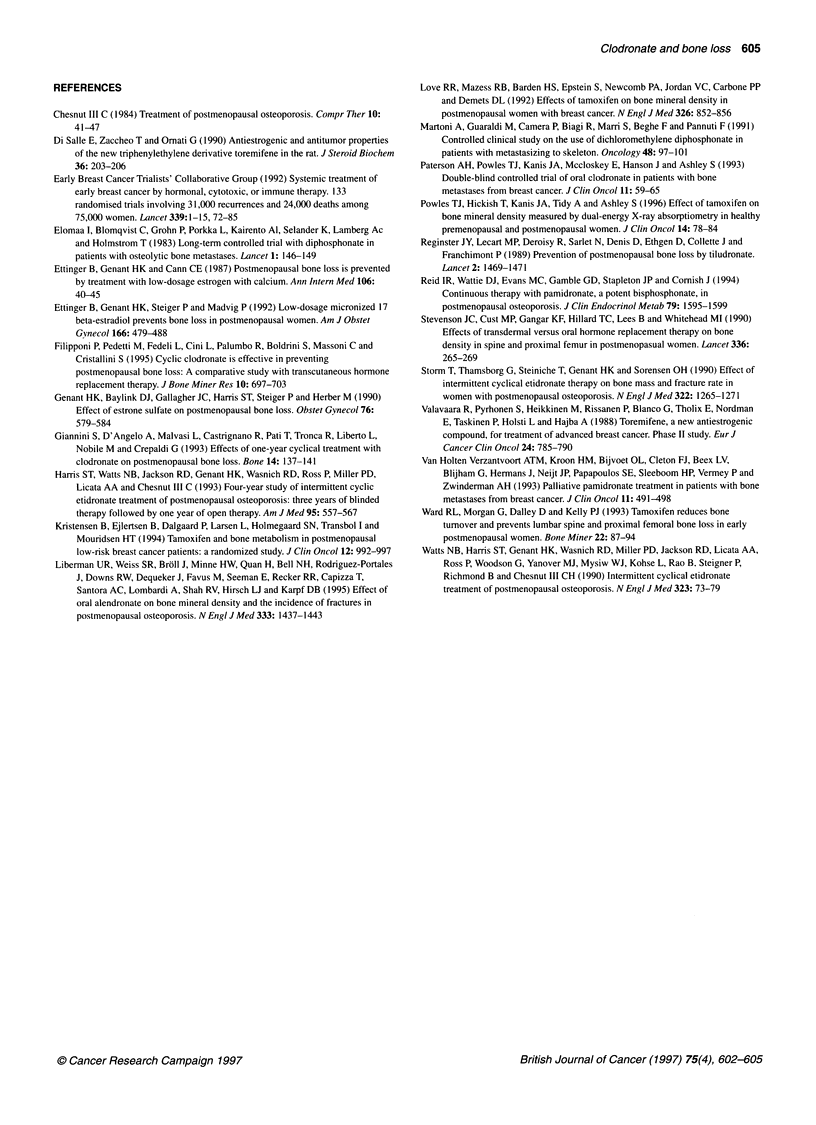# Clodronate improves bone mineral density in post-menopausal breast cancer patients treated with adjuvant antioestrogens.

**DOI:** 10.1038/bjc.1997.105

**Published:** 1997

**Authors:** T. Saarto, C. Blomqvist, M. Välimäki, P. Mäkelä, S. Sarna, I. Elomaa

**Affiliations:** Department of Oncology, Helsinki University Central Hospital, Finland.

## Abstract

The effect of clodronate on bone mineral density (BMD) was studied in 121 post-menopausal breast cancer women without skeletal metastases. In addition, two antioestrogens, tamoxifen and toremifene, were compared in their action on bone mineral density. Patients were randomized to have an adjuvant antioestrogen treatment either 20 mg of tamoxifen or 60 mg of toremifene daily for 3 years. In addition all patients were randomized to have 1600 mg of oral clodronate daily or to act as control subjects. BMD of the lumbar spine and femoral neck were measured by dual-energy radiographic absorptiometry before therapy and at 1 and 2 years. At 2 years, clodronate with antioestrogens markedly increased BMD in the lumbar spine and femoral neck by 2.9% and 3.7% (P = 0.001 and 0.006 respectively). There were no significant changes in BMD in the patients given antioestrogens only. No significant differences were found between tamoxifen and toremifene on bone mineral density. Clodronate with antioestrogens significantly increased bone mass in the lumbar spine and femoral neck. Both antioestrogens, tamoxifen and toremifene, similarly prevented bone loss in the lumbar spine and femoral neck.


British Journal of Cancer (1997) 75(4), 602–605
© 1997 Cancer Research Campaign

### Clodronate improves bone mineral density in post-menopausal breast cancer patients treated with adjuvant antioestrogens

T Saarto[1], C Blomqvist[1], M Välimäki[2], P Mäkelä[3], S Sarna[4] and I Elomaa[1]

[1]Department of Oncology, Helsinki University Central Hospital; [2]Department of Medicine, Helsinki University Hospital; [3]Department of Diagnostic Radiology, Helsinki University Hospital; [4]Department of Public Health (Biostatistics), University of Helsinki, Helsinki, Finland

**Summary** The effect of clodronate on bone mineral density (BMD) was studied in 121 post-menopausal breast cancer women without skeletal metastases. In addition, two antioestrogens, tamoxifen and toremifene, were compared in their action on bone mineral density. Patients were randomized to have an adjuvant antioestrogen treatment either 20 mg of tamoxifen or 60 mg of toremifene daily for 3 years. In addition all patients were randomized to have 1600 mg of oral clodronate daily or to act as control subjects. BMD of the lumbar spine and femoral neck were measured by dual-energy radiographic absorptiometry before therapy and at 1 and 2 years. At 2 years, clodronate with antioestrogens markedly increased BMD in the lumbar spine and femoral neck by 2.9% and 3.7% ($P = 0.001$ and 0.006 respectively). There were no significant changes in BMD in the patients given antioestrogens only. No significant differences were found between tamoxifen and toremifene on bone mineral density. Clodronate with antioestrogens significantly increased bone mass in the lumbar spine and femoral neck. Both antioestrogens, tamoxifen and toremifene, similarly prevented bone loss in the lumbar spine and femoral neck.

**Keywords:** antioestrogens; bone mineral density; bisphosphonate; breast neoplasm; post-menopausal osteoporosis; toremifene

Adjuvant antioestrogen treatment with tamoxifen significantly improves the survival of post-menopausal women with primary breast cancer (EBCTCG, 1992). Tamoxifen has oestrogen agonistic effects on bone and therefore prevents bone loss in post-menopausal women (Love et al, 1992; Ward et al, 1993; Kristensen et al, 1994; Powles et al, 1996). Tamoxifen has been shown to prevent bone loss predominantly in the lumbar spine (Love et al, 1992; Kristensen et al, 1994), but in two randomized studies this was also true for the upper femur (Ward et al, 1993; Powles et al, 1996).

Toremifene is a close analogue to tamoxifen with demonstrated efficacy in advanced breast cancer (Valavaara et al, 1988). Compared with tamoxifen, toremifene is more oestrogen antagonistic than agonistic in rat (Di Salle et al, 1990). At present, no data are available on the effect of toremifene on bone.

Since bisphosphonates prevent post-menopausal bone loss (Chesnut 1984; Reginster et al, 1989; Storm et al, 1990; Watts et al, 1990; Giannini et al, 1993; Harris et al, 1993; Reid et al, 1994; Filipponi et al, 1995; Liberman et al, 1995) and in advanced breast cancer reduced the development of new bone metastases (Elomaa et al, 1983; Martoni et al, 1991; Paterson et al, 1993; Van Holten Verzantvoort et al, 1993), they are attractive candidates for the treatment of patients with early breast cancer.

We performed a prospective, open, randomized study to determine the effect of oral clodronate in post-menopausal women with primary breast cancer treated with adjuvant antioestrogens, tamoxifen or toremifene. In addition, tamoxifen and toremifene were compared in their action on bone mineral density.

Received 24 July 1996
Revised 10 October 1996
Accepted 15 October 1996

Correspondence to: I Elomaa, Department of Oncology, Helsinki University Central Hospital, Haartmaninkatu 4, FIN-00290 Helsinki, Finland

### MATERIALS AND METHODS

### Patients and methods

The study population consisted of 121 early-stage breast cancer patients, with no haematogenic metastases. Eligible for the trial were post-menopausal women with operable breast cancer and histologically proven axillary metastases, T1–3 N1–2 M0, treated between May 1992 and July 1993 at Helsinki University Hospital, Department of Oncology. Exclusion criteria included the following: (1) age above 75 years; (2) Karnofsky performance index below 70%; (3) bone metastases within 6 months after BMD measurement; (4) other malignancies; (5) peptic ulcer or its symptoms; and (6) creatinine over 150 $\mu mol\ l^{-1}$. Post-menopausal status at entry was defined as either no menses for more than 1 year or shorter duration of amenorrhoea with follicle-stimulating hormone (FSH) levels in the post-menopausal range.

All patients underwent surgery with axillary clearance and total mastectomy or breast-conserving resection. All patients also had post-operative radiotherapy with megavoltage irradiation (50 Gy in 25 fractions) to regional lymph nodes and operative scar or remaining breast after breast-conserving resection, simultaneously with adjuvant therapy. Patients were randomly allocated to receive adjuvant antioestrogen therapy: 20 mg of tamoxifen per day or 60 mg of toremifene daily for 3 years. In addition, all patients were randomized to receive or not to receive 1600 mg of oral clodronate (Bonefos, Leiras) daily for 3 years.

Informed consent was obtained from all participants. The study was approved by the local ethics committee at the Department of Oncology. Helsinki University Hospital. Staging investigations for breast cancer included clinical investigation, liver ultrasound, chest radiograph and bone scintigraphy. Basic laboratory tests before randomization included a complete blood count and sedimentation rate, liver enzymes (transaminase, alkaline phosphatase and 5-nucleotidase), serum creatinine, calcium and electrolytes. Patients were interviewed regarding menopausal status, medications and other diseases before randomization and every 12 months. Bone scintigraphy and measurements of serum FSH luteinizing hormone (LH) and oestradiol were performed before treatment and every 12 months thereafter. Plasma concentrations of FSH and LH were measured by immunofluorometric assays (IFMA; Wallac, Turku, Finland) and plasma oestradiol levels were measured by a radioimmunoassay (RIA; Farmos, Oulunsalo, Finland). Clinical investigation and basic laboratory safety tests were repeated every 4 months with a radiological examination if necessary.

### Bone densitometry

Bone mineral density (BMD, g $cm^{-2}$) was measured by dual-energy radiographic absorptiometry (DXA) using a Hologic QDR-1000 densitometer (Hologic Waltham, MA, USA). BMD was measured at the lumbar vertebrae (L1–L4) and femoral neck in the right femoral area before the initiation of therapy and every 12 months. The coefficient of variation for the precision of the BMD measurements in the lumbar vertebrae and femoral neck was 0.9% and 1.2% respectively.

**Table 1** Pretreatment characteristics [mean and (s.d.), median and range, or absolute number and percentage] of patients in clodronate and control groups

| | Clodronate | | Control | |
|---|---|---|---|---|
| Number of patients | 44 | | 49 | |
| Age years | 61 | (7) | 62 | (7) |
| Weight (kg) | 67 | (8) | 71 | (12) |
| Height (cm) | 163 | (6) | 163 | (5) |
| Body mass index | 25.3 | (3.6) | 26.7 | (4.4) |
| FSH (U $l^{-1}$) | 50.9 | 3.8–100.1 | 58.9 | 14.6–109.0 |
| LH (U $l^{-1}$) | 35.9 | 2.8–89.2 | 36.0 | 10.8–94.5 |
| Oestradiol (nmol $l^{-1}$) | 0.02 | 0.01–0.43 | 0.02 | 0.02–0.28 |
| Lumbar spine BMD | 0.905 | (0.140) | 0.952 | (0.136) |
| Femoral neck BMD | 0.735 | (0.101) | 0.774 | (0.128) |
| Karnofsky (%) | | | | |
| 100 | 31 | 70% | 40 | 82% |
| 90–80 | 13 | 30% | 9 | 18% |
| Operation | | | | |
| Mastectomy | 33 | 75% | 33 | 67% |
| Lumpectomy | 11 | 25% | 16 | 33% |
| T | | | | |
| T1 | 22 | 50% | 28 | 57% |
| T2 | 15 | 34% | 18 | 37% |
| T3 | 5 | 11% | 2 | 4% |
| Unknown | 2 | 5% | 1 | 2% |
| N | | | | |
| N (1–3) | 32 | 73% | 37 | 76% |
| N (4–10) | 10 | 23% | 10 | 20% |
| N (>10) | 1 | 2% | 2 | 4% |
| | 1 | 2% | 0 | |
| Histology | | | | |
| Ductal | 34 | 77% | 43 | 88% |
| Lobular | 10 | 23% | 5 | 10% |
| Other | 0 | 0% | 1 | 2% |
| Oestrogen receptors | | | | |
| Positive | 31 | 70% | 37 | 76% |
| Negative | 10 | 23% | 8 | 16% |
| Unknown | 3 | 7% | 4 | 8% |
| Progesterone receptors | | | | |
| Positive | 22 | 50% | 30 | 61% |
| Negative | 19 | 43% | 15 | 31% |
| Unknown | 3 | 7% | 4 | 8% |

### Statistical methods

BMD values are expressed as a percentage of the baseline value. The effect of treatments (clodronate and antioestrogens) on changes in BMD at 1 and 2 years was tested by a repeated measures ANOVA model using the BMDP2V programs (BMDP Release 7), with change from baseline BMD as the dependent variable, antioestrogen treatment and clodronate treatment as the grouping variables. Other comparisons were made using the Mann–Whitney test or Wilcoxon matched pair test. Confidence intervals (95%) were calculated for the main outcome measures.

### RESULTS

Pretreatment characteristics of the subjects in the two study groups are given in Table 1. The two groups were well balanced with respect to pretreatment characteristics, previous diseases and medications. There were no severe abnormalities in baseline laboratory tests. None of the patients had previously used bisphosphonates or calcitonin.

Of the 121 eligible patients, data from 28 patients were excluded from the analyses: 16 owing to metastatic disease, nine as a result of protocol violation (patients treated with chemotherapy) and there because of diseases affecting calcium and bone metabolism.

**Figure 1** Changes from baseline and 95% confidence intervals in BMD of (**A**) the lumbar spine and (**B**) femoral neck at 1 and 2 years in the control and clodronate groups. Clodronate group -■-, control group -□-

© Cancer Research Campaign 1997

**Table 2** Percentage changes (mean and 95% confidence intervals) from baseline in BMD of the lumbar spine and femoral neck at 1 and 2 years in the tamoxifen and toremifene groups

| Treatment groups | Mean change % (CI) at 1 year | | Mean change % (CI) at 2 years | |
|---|---|---|---|---|
| | Control | Clodronate | Control | Clodronate |
| *Lumbar spine* | | | | |
| Tamoxifen | –0.3<br>(–1.9 to +1.3) | +1.7<br>(0 to +3.4) | –1.5<br>(–3.4 to +0.5) | +2.5<br>(+0.6 to +4.5) |
| Toremifene | +0.6<br>(–0.8 to +2.0) | +1.1<br>(+0.3 to +1.8) | +0.5<br>(–1.6 to +2.6) | +3.4<br>(+0.9 to +5.9) |
| *Femoral neck* | | | | |
| Tamoxifen | +1.0<br>(–0.9 to +2.9) | +3.9<br>(+2.2 to +5.6) | +2.0<br>(–0.6 to +4.5) | +4.2<br>(+2.2 to +6.1) |
| Toremifene | +0.4<br>(–2.2 to +3.0) | +2.6<br>(+0.3 to +4.8) | –0.3<br>(–3.3 to +1.2) | +3.0<br>(+0.5 to +5.5) |

The number of patients treated with tamoxifen only, toremifene only, tamoxifen with clodronate, and toremifene with clodronate are 23, 21, 23 and 16, respectively, at both 1 and 2 years.

Thus, 93 patients were eligible for analyses. From the 2 years' analyses, nine additional patients were excluded: eight because of breast cancer recurrence and one patients because of discontinuation of follow-up.

Four patients interrupted clodronate treatment: three patients because of side-effects and one patient because of refusal to continue therapy. One patient had a dose reduction because of gastric pain. Five patients interrupted antioestrogen therapy after a median of 10 months: four because of menopausal symptoms and one because of increased serum transaminase levels. All these patients are included in the analyses.

Oral clodronate treatment was well tolerated. Three patients (3%) interrupted clodronate therapy and one patient reduced the dose because of gastric pain. There were no significant differences in adverse events between the study groups. During the first year of therapy, seven patients (8%) reported mild nausea and vomiting, and three patients (3%) had diarrhoea with no differences between the study groups. During the second year four (4%) patients complained about of mild nausea and vomiting, and one patient had diarrhoea. Renal toxicity was not seen in either group.

### Hormonal changes

Antioestrogen therapy decreased FSH and LH levels significantly in post-menopausal patients: median FSH from 54.6 U $l^{-1}$ (range 3.8–109) to 29.9 U $l^{-1}$ (9.0–66.1), median LH from 36.0 U $l^{-1}$ (2.8–94.5) to 20.0 U $l^{-1}$ (4.4–62.9) ($P < 0.0001$ and $P < 0.0001$ respectively, Wilcoxon), with no significant changes in oestradiol levels. There were no significant differences between the tamoxifen and toremifene groups, or between the clodronate and control groups.

The mean weight gain was 1.4 kg (s.d. 2.8) and 1.4 kg (s.d. 4.5) at 1 and 2 years of the study ($P < 0.0001$ and $P < 0.0001$ respectively, Wilcoxon); there were no significant differences between the control and clodronate groups, nor between the tamoxifen and toremifene groups.

### Changes in bone mineral density

The baseline values for the BMD of the lumbar spine and the femoral neck were similar in the clodronate and control groups (Table 1). In patients receiving clodronate, BMD of the lumbar spine increased significantly by 1.5% and 2.9% at 1 and 2 years respectively (within the clodronate group, $P$ = 0.002, and $P$ = 0.002, Wilcoxon), while in the control group BMD of the lumbar spine was unchanged (between the groups, $P$ = 0.004, ANOVA). BMD of the femoral neck increased by 3.3% and 3.7% in the clodronate group at the first and second years (within the clodronate group, $P < 0.0001$ and $P < 0.0001$, Wilcoxon), with no significant changes in the control group (between the groups, $P$ = 0.003, ANOVA) (Figure 1).

Changes of BMD in the tamoxifen and toremifene groups are shown in Table 2. The type of endocrine treatment had no significant effect on the BMD of the lumbar spine and femoral neck (at 2 years, $P$ = 0.446 and $P$ = 0.064 respectively).

### DISCUSSION

The present study implies that adjuvant antioestrogen treatment with either tamoxifen or toremifene prevents bone loss in post-menopausal women. However, bone mineral density can even be augmented by combining clodronate with the medical treatment of post-menopausal women with breast cancer.

In line with prior investigations, post-menopausal breast cancer patients treated with antioestrogens did not lose bone at the lumbar spine or at the femoral neck (Love et al, 1992; Ward et al, 1993; Kristensen et al, 1994; Powles et al, 1996). Previous data on tamoxifen were extended by the finding that a novel antioestrogen, toremifene, preserved bone mass as effectively as tamoxifen, although experimental data have shown it to be less oestrogenic than tamoxifen (Di Salle et al, 1990). Clodronate provided an additional benefit for post-menopausal breast cancer patients. In fact, the clodronate-induced increase in bone mass was quite similar to that obtained on oestrogen replacement therapy after a natural menopause (Ettinger et al, 1987, 1992; Genant et al, 1990; Stevenson et al, 1990).

Our results indicate that clodronate combined with antioestrogens augment bone mass in post-menopausal women. A new antioestrogen, toremifene, had an oestrogen-like effect on bone similar to tamoxifen. Since clodronate can also inhibit the development of bone metastases, it appears to be an attractive adjuvant treatment for women with breast cancer.

© Cancer Research Campaign 1997

### REFERENCES

Chesnut III C (1984) Treatment of postmenopausal osteoporosis. *Compr Ther* **10:** 41–47

Di Salle E, Zaccheo T and Ornati G (1990) Antiestrogenic and antitumor properties of the new triphenylethylene derivative toremifene in the rat. *J Steroid Biochem* **36:** 203–206

Early Breast Cancer Trialists' Collaborative Group (1992) Systemic treatment of early breast cancer by hormonal, cytotoxic, or immune therapy. 133 randomised trials involving 31,000 recurrences and 24,000 deaths among 75,000 women. *Lancet* **339:**1–15, 72–85

Elomaa I, Blomqvist C, Grohn P, Porkka L, Kairento Al, Selander K, Lamberg Ac and Holmstrom T (1983) Long-term controlled trial with diphosphonate in patients with osteolytic bone metastases. *Lancet* **1:** 146–149

Ettinger B, Genant HK and Cann CE (1987) Postmenopausal bone loss is prevented by treatment with low-dosage estrogen with calcium. *Ann Intern Med* **106:** 40–45

Ettinger B, Genant HK, Steiger P and Madvig P (1992) Low-dosage micronized 17 beta-estradiol prevents bone loss in postmenopausal women. *Am J Obstet Gynecol* **166:** 479–488

Filipponi P, Pedetti M, Fedeli L, Cini L, Palumbo R, Boldrini S, Massoni C and Cristallini S (1995) Cyclic clodronate is effective in preventing postmenopausal bone loss: A comparative study with transcutaneous hormone replacement therapy. *J Bone Miner Res* **10:** 697–703

Genant HK, Baylink DJ, Gallagher JC, Harris ST, Steiger P and Herber M (1990) Effect of estrone sulfate on postmenopausal bone loss. *Obstet Gynecol* **76:** 579–584

Giannini S, D'Angelo A, Malvasi L, Castrignano R, Pati T, Tronca R, Liberto L, Nobile M and Crepaldi G (1993) Effects of one-year cyclical treatment with clodronate on postmenopausal bone loss. *Bone* **14:** 137–141

Harris ST, Watts NB, Jackson RD, Genant HK, Wasnich RD, Ross P, Miller PD, Licata AA and Chesnut III C (1993) Four-year study of intermittent cyclic etidronate treatment of postmenopausal osteoporosis: three years of blinded therapy followed by one year of open therapy. *Am J Med* **95:** 557–567

Kristensen B, Ejlertsen B, Dalgaard P, Larsen L, Holmegaard SN, Transbol I and Mouridsen HT (1994) Tamoxifen and bone metabolism in postmenopausal low-risk breast cancer patients: a randomized study. *J Clin Oncol* **12:** 992–997

Liberman UR, Weiss SR, Bröll J, Minne HW, Quan H, Bell NH, Rodriguez-Portales J, Downs RW, Dequeker J, Favus M, Seeman E, Recker RR, Capizza T, Santora AC, Lombardi A, Shah RV, Hirsch LJ and Karpf DB (1995) Effect of oral alendronate on bone mineral density and the incidence of fractures in postmenopausal osteoporosis. *N Engl J Med* **333:** 1437–1443

Love RR, Mazess RB, Barden HS, Epstein S, Newcomb PA, Jordan VC, Carbone PP and Demets DL (1992) Effects of tamoxifen on bone mineral density in postmenopausal women with breast cancer. *N Engl J Med* **326:** 852–856

Martoni A, Guaraldi M, Camera P, Biagi R, Marri S, Beghe F and Pannuti F (1991) Controlled clinical study on the use of dichloromethylene diphosphonate in patients with metastasizing to skeleton. *Oncology* **48:** 97–101

Paterson AH, Powles TJ, Kanis JA, Mccloskey E, Hanson J and Ashley S (1993) Double-blind controlled trial of oral clodronate in patients with bone metastases from breast cancer. *J Clin Oncol* **11:** 59–65

Powles TJ, Hickish T, Kanis JA, Tidy A and Ashley S (1996) Effect of tamoxifen on bone mineral density measured by dual-energy X-ray absorptiometry in healthy premenopausal and postmenopausal women. *J Clin Oncol* **14:** 78–84

Reginster JY, Lecart MP, Deroisy R, Sarlet N, Denis D, Ethgen D, Collette J and Franchimont P (1989) Prevention of postmenopausal bone loss by tiludronate. *Lancet* **2:** 1469–1471

Reid IR, Wattie DJ, Evans MC, Gamble GD, Stapleton JP and Cornish J (1994) Continuous therapy with pamidronate, a potent bisphosphonate, in postmenopausal osteoporosis. *J Clin Endocrinol Metab* **79:** 1595–1599

Stevenson JC, Cust MP, Gangar KF, Hillard TC, Lees B and Whitehead MI (1990) Effects of transdermal versus oral hormone replacement therapy on bone density in spine and proximal femur in postmenopasual women. *Lancet* **336:** 265–269

Storm T, Thamsborg G, Steiniche T, Genant HK and Sorensen OH (1990) Effect of intermittent cyclical etidronate therapy on bone mass and fracture rate in women with postmenopausal osteoporosis. *N Engl J Med* **322:** 1265–1271

Valavaara R, Pyrhonen S, Heikkinen M, Rissanen P, Blanco G, Tholix E, Nordman E, Taskinen P, Holsti L and Hajba A (1988) Toremifene, a new antiestrogenic compound, for treatment of advanced breast cancer. Phase II study. *Eur J Cancer Clin Oncol* **24:** 785–790

Van Holten Verzantvoort ATM, Kroon HM, Bijvoet OL, Cleton FJ, Beex LV, Blijham G, Hermans J, Neijt JP, Papapoulos SE, Sleeboom HP, Vermey P and Zwinderman AH (1993) Palliative pamidronate treatment in patients with bone metastases from breast cancer. *J Clin Oncol* **11:** 491–498

Ward RL, Morgan G, Dalley D and Kelly PJ (1993) Tamoxifen reduces bone turnover and prevents lumbar spine and proximal femoral bone loss in early postmenopausal women. *Bone Miner* **22:** 87–94

Watts NB, Harris ST, Genant HK, Wasnich RD, Miller PD, Jackson RD, Licata AA, Ross P, Woodson G, Yanover MJ, Mysiw WJ, Kohse L, Rao B, Steigner P, Richmond B and Chesnut III CH (1990) Intermittent cyclical etidronate treatment of postmenopausal osteoporosis. *N Engl J Med* **323:** 73–79

*© Cancer Research Campaign 1997*